# Functional genomic effects of indels using Bayesian genome-phenome wide association studies in sorghum

**DOI:** 10.3389/fgene.2023.1143395

**Published:** 2023-03-31

**Authors:** J. Lucas Boatwright, Sirjan Sapkota, Stephen Kresovich

**Affiliations:** ^1^ Department of Plant and Environmental Sciences, Clemson University, Clemson, SC, United States; ^2^ Advanced Plant Technology, Clemson University, Clemson, SC, United States; ^3^ Feed the Future Innovation Lab for Crop Improvement, Cornell University, Ithaca, NY, United States

**Keywords:** genome-phenome wide association, bayesian regression, insertions, deletions, sorghum, alphafold

## Abstract

High-throughput genomic and phenomic data have enhanced the ability to detect genotype-to-phenotype associations that can resolve broad pleiotropic effects of mutations on plant phenotypes. As the scale of genotyping and phenotyping has advanced, rigorous methodologies have been developed to accommodate larger datasets and maintain statistical precision. However, determining the functional effects of associated genes/loci is expensive and limited due to the complexity associated with cloning and subsequent characterization. Here, we utilized phenomic imputation of a multi-year, multi-environment dataset using *PHENIX* which imputes missing data using kinship and correlated traits, and we screened insertions and deletions (InDels) from the recently whole-genome sequenced Sorghum Association Panel for putative loss-of-function effects. Candidate loci from genome-wide association results were screened for potential loss of function using a Bayesian Genome-Phenome Wide Association Study (BGPWAS) model across both functionally characterized and uncharacterized loci. Our approach is designed to facilitate *in silico* validation of associations beyond traditional candidate gene and literature-search approaches and to facilitate the identification of putative variants for functional analysis and reduce the incidence of false-positive candidates in current functional validation methods. Using this Bayesian GPWAS model, we identified associations for previously characterized genes with known loss-of-function alleles, specific genes falling within known quantitative trait loci, and genes without any previous genome-wide associations while additionally detecting putative pleiotropic effects. In particular, we were able to identify the major tannin haplotypes at the *Tan1* locus and effects of InDels on the protein folding. Depending on the haplotype present, heterodimer formation with *Tan2* was significantly affected. We also identified major effect InDels in *Dw2* and *Ma1*, where proteins were truncated due to frameshift mutations that resulted in early stop codons. These truncated proteins also lost most of their functional domains, suggesting that these indels likely result in loss of function. Here, we show that the Bayesian GPWAS model is able to identify loss-of-function alleles that can have significant effects upon protein structure and folding as well as multimer formation. Our approach to characterize loss-of-function mutations and their functional repercussions will facilitate precision genomics and breeding by identifying key targets for gene editing and trait integration.

## 1 Introduction

Genome-Wide Association Studies (GWAS) have served as powerful tools for genotype-phenotype mapping of genomic regions that can be used as breeding targets since their initial application (Ozaki et al., 2002). As newer models have been developed, GWAS have seen significant improvements in computational efficiency (Zhou and Stephens, 2012), statistical power (Liu et al., 2016; Huang et al., 2019), user-friendly interfaces (Yin et al., 2021), and development of multi-response regression techniques (Zhou and Stephens, 2014). However, as the scale of genomic and phenomic data continues to grow, both traditional and newer tools will be required to make the best use of massive biological datasets (Bilder et al., 2009). For advocates of phenomic approaches, the expectation is that the broad-scale study of multiscale phenotypes will better dissect complex genetic architecture and subtly correlated biological networks (Boatwright et al., 2022b). As phenomic data increasingly capture orthogonal or partially correlated traits, it will be increasingly feasible to functionally characterize and improve multiple traits simultaneously in the breeding process (Eberius and Lima-Guerra, 2009).

The decreased costs of high-throughput sequencing have led to the exponential growth of genomic data (Furbank and Tester, 2011) including single-nucleotide polymorphisms (SNPs), insertions and deletions (InDels), and copy-number variants (CNVs). Due to costs associated with acquiring these data, the technical complexity of generating InDels and CNVs, and the ability of SNPs to estimate relatedness and find associated loci, SNPs are the most prevalent variant types studied with InDels and CNVs being largely underrepresented among genomic studies. Conversely, phenomic data acquisition has lagged due to several complications. These complications include, but are not limited to, 1) manual collection of data for validation, 2) flexibility (e.g., across crops, architecture, conditions, etc.) and costs (e.g., high-performance liquid chromatography) of high-throughput technologies, 3) interoperability for phenotyping under both control and field conditions, 4) data integration, management, and modelling, and 5) limited options for informatic tools and resources (Houle et al., 2010; Araus et al., 2018). As such, collection and processing of phenomic data have been major bottlenecks for genotype-phenotype mapping, and for most biological systems, those data have been collected over many years rather than at scale by a few studies (Furbank and Tester, 2011).

Once phenomic data are collected, genotype-phenotype mapping typically occurs using either quantitative trait loci (QTL) mapping (Broman et al., 2019) or GWAS approaches (Zhou and Stephens, 2012; Yin et al., 2021). Unfortunately, as these methods are susceptible to false positives - due to the extent of multiple testing and effects of population stratification - downstream validation of associations *via* tissue culture and genetic transformation represent pivotal steps in the complete characterization of novel loci. Even in maize, characterization *via* tissue culture involves considerable effort. Both transformation and gene editing are limited in scale by the work required for tissue culture and plant regeneration, which takes several months (Ishida et al., 2020). As such, annotation and validation of putatively novel loci represent additional major bottlenecks for advances in basic science and adoption of novel findings in a given research program. Just as data integration, management, and modelling are complicating factors for phenomics, limited options for informatic tools and resources place an additional burden for research groups with viable phenomic datasets. Limitations for standardized trait ontologies and databases further compound problems, especially for researchers working with non-model or recently sequenced organisms (Bilder et al., 2009).

Here, we implement a Bayesian Genome-Phenome Wide Association Study (BGPWAS) model that offers improvements over existing genotype-phenotype mapping approaches in its ability to identify pleiotropic and putative knockdown/out effects of given loci *in silico* (Liang et al., 2020). This model overcomes several limitations of traditional frequentist approaches to provide clearer and better targeted results. In particular, as this approach may require potentially thousands of statistical test, the use of strong regularizing priors is of vital importance for controlling false-positive results (Bilder et al., 2009). Additionally, this model may be run with SNP, InDel, CNV, or haplotype data as necessary to accommodate various use cases. As larger mutations are more likely to exhibit functional effects, InDels and CNVs provide an increased probability to identify functionally relevant mutations. Once putatively functional mutations are identified, we apply AlphaFold’s neural network-based model to predict protein structures at scale (Jumper et al., 2021). Prediction of protein structures provides the opportunity to perform *in silico* validation of mutations quickly, at scale, and as a means to screen association results before moving to resource-intensive tissue culture methods. AlphaFold is further capable of predicting both monomer and multimer formation, which provides the unique opportunity to examine protein-protein interactions across protein isoforms and better understand the genetic networks underpinning associated phenotypes (Jumper et al., 2021). By performing *in silico* validation of potential mutations to identify their functional effects, our approach can accurately identify novel targets for altering gene expression and integration of key traits of interest in plant breeding. We expect that our statistical approach will improve phenotypic characterization of genes through joint consideration of genomic and phenomic data.

To demonstrate the value of these approaches, we apply these models to the Sorghum Association Panel (SAP) (Casa et al., 2008). The SAP is composed of both temperate-adapted breeding lines and converted (photoperiod-insensitive) tropical accessions from the Sorghum Conversion Program (SCP) (Stephens et al., 1967; Klein et al., 2008). Accessions were selected to maximize the genetic and phenotypic diversity of the panel while simultaneously capturing accessions with significant demographic history and historical breeding importance (Casa et al., 2008). The recent whole-genome resequencing of the SAP included 400 individuals and identified approximately 5.4 million SNPs, 2.6 million InDels, and 170,000 CNVs after quality filtering (Boatwright et al., 2022a). Due to the high quality and scale of these genomic data, we utilized this resource here. In sorghum, plant height and tannin content represent vital phenotypes due to both historic selection (Wu et al., 2019) and modern agriculture (Dillon et al., 2007). As an important domestication trait, tannin content has been shown to lower nutrient uptake (Xiong et al., 2019). Conversely, phenolic compounds like tannins can limit pest damage due to their bitter flavor (Wu et al., 2019), exhibit antimicrobial properties (Shields et al., 2021), and tannin antioxidant activities can also improve gut health (Xiong et al., 2019). As such, we focus on several known and putatively novel loci mediating these traits.

## 2 Materials and methods

### 2.1 Phenomic data and imputation

Phenomic data constituting 234 traits measured on the SAP (Casa et al., 2008) were obtained from Mural et al. (2021). Traits were filtered such that any trait with more than 30% missing data were removed from subsequent analyses. The 30% threshold was selected to reduce overall missingness while simultaneously reducing the impact of imputation on the final results and maintaining at least 100 traits, as Liang et al., 2020 indicated that model power significantly increased with increasing feature count. Filtering at a 30% threshold for each trait resulted in a total of only 11% missing data across the remaining 124 traits, which was lowered to 10% after removing 10 individuals not represented in the genomic data. These traits represented a mixture of four agronomic, 29 biochemical, five disease, 27 reproductive, 10 root, 18 seed, and 31 vegetative traits. For phenotypic imputation of the filtered traits, we used PHENotype Imputation eXpediated, (PHENIX) (Dahl et al., 2016), which imputes a matrix of partially observed phenotypes, Y, (an N×p matrix of N individuals row-wise and p phenotypes column-wise) that have been centered and scaled. A standard Multiple Phenotype Mixed Model (MPMM) has the form,

Y=U+ϵ (1)

where U is an N×*p* matrix of random effects and *ϵ* is a N×*p* matrix of residuals and are modeled using matrix Gaussian distributions as follows

$$\begin{array}{cc} \;U & ∼MN\left(0,K,B\right),\\ \;\epsilon & ∼MN\left(0,I_{N},E\right) \end{array}$$ (2)

In this model K is the N×N kinship matrix between accessions (or row-wise covariance), B is the p×p matrix of genetic covariances between phenotypes (or column-wise covariance), E is the p×p matrix of residual covariances between phenotypes, and *I*_*N*_ is the identity matrix of size N×N. PHENIX uses a Bayesian MPMM to fit a low-rank model for U, such that U = S *β*, where

$$\begin{array}{cc} \;S & ∼MN\left(0,K,I_{P}\right),\\ \;\beta & ∼MN\left(0,I_{P},\tau^{-1}I_{P}\right), \end{array}$$ (3)

Where *MN* represents a matrix normal distribution with mean zero, *I*_*P*_ is an identity matrix of size p×p, *τ* is a regularization parameter, and a Wishart prior (*Wi*) is used for the residual precision matrix *E* ^−1^

$$\;E^{-1}∼Wi\left(P+5,\frac{1}{4}I_{P}\right)$$ (4)

where the prior has *p* + 5 degrees of freedom and scale $\frac{1}{4}I_{P}$ .

The model is fit using Variational Bayes methods resulting in sample posteriors with multivariate normal distributions, and missing data are imputed using the posterior mean. In summary, PHENIX uses known kinship and trait covariance to better predict missing phenotypic data (Dahl et al., 2016). As the original PHENIX did not work properly on R v4.1.0, we also provide a slightly modified version used in the study on GitHub (https://github.com/jlboat/PHENIX).

### 2.2 Genomic data

Genomic data were obtained and processed as described in (Boatwright et al., 2022a). In short, 30x whole-genome sequencing was performed using an Illumina NovaSeq 6,000 sequencer resulting in paired-end 150-bp reads for 400 SAP accessions, and variants were called against the BTx623 version 3.1.1 annotated reference genome (McCormick et al., 2018) using the GATK variant calling pipeline (McKenna et al., 2010) and best practices (DePristo et al., 2011; Van der Auwera et al., 2013). The resulting variants were quality filtered to reduce false positives (MAF < 0.1, QD < 2, InbreedingCoeff < 0, QUAL < 30, SOR > 3, FS > 60, MQ < 40, MQRankSum < -12.5, and ReadPosRankSum < -8) (DePristo et al., 2011; Danecek et al., 2021) and imputed using Beagle (Browning et al., 2018). Principal components (PCs) were obtained using both SNPs and InDels based on a leave-one-chromosome-out (LOCO) approach (Yang et al., 2014) to prevent confounding of components with the response variant’s chromosome of origin. The LOCO PCs were calculated using Plink v1.90b6.10 (Purcell et al., 2007). While all PCs were estimated, only the top three PCs were used for each model run as this number has been shown to account for population structure in previous sorghum studies (Boatwright et al., 2022a). The number of PCs may be altered as necessary. For the current study, we isolated both insertions and deletions (InDels) from the full set of imputed genomic data resulting in 1,349,015 InDels. The BGPWAS was executed sequentially on each InDel for a given gene using custom scripts (https://github.com/jlboat/BGPWAS) using the model described below.

### 2.3 Bayesian genome-phenome wide association model

Our Bayesian GPWAS was executed using stan (Stan Development Team, 2019) and rstanarm (Goodrich et al., 2022), which uses the No-U-Turn-Sampler (NUTS) as the default Markov Chain Monte Carlo (MCMC) sampler (Hoffman and Gelman, 2014). In brief, the NUTS does not perform a random walk of the parameter space thereby circumventing sensitivity to correlated parameters characteristic of many MCMC methods. Instead, the NUTS uses first-order gradient information to inform which steps should be taken, allowing the sample posteriors to converge on high-dimensional target distributions much more quickly than simpler methods such as random walk Metropolis or Gibbs sampling (Hoffman and Gelman, 2014). The Bayesian GPWAS model has the form,

$$\begin{array}{cc} \;y_{i} & =\beta X_{i}+\epsilon_{i},\\ \;\epsilon_{i} & ∼N\left(0,\sigma^{2}\right), \end{array}$$ (5)

for i = 1, … , n, where n is the number of samples. The response variable *y* represents numerically encoded variant data (e.g. SNPs, Indels), *X* represents the matrix of predictors including traits (124) and LOCO-based principal components 3) as described above (see Genomic Data), and *ϵ* represents the residual variance. Coefficients are estimated using a horseshoe prior (Piironen and Vehtari, 2017c) where,

$$\begin{array}{cc} \;\beta_{j}|\lambda_{j},\tau & ∼N\left(0,{\lambda_{j}}^{2}\tau^{2}\right),\\ \;\lambda_{j} & ∼C^{+}\left(0,1\right), \end{array}$$ (6)

for j = 1, … , D. The global regularization parameter *τ* shrinks all *β*_*j*_ toward zero, while the local parameters *λ*_*j*_ allow some *β*_*j*_ to escape the shrinkage through the heavy-tailed half-Cauchy prior (Piironen and Vehtari, 2017c). The effective number of non-zero coefficients is controlled using a user defined *τ*, which we scale based upon the number of predictors and sample size as follows:

$$\;\tau_{0}=\frac{p_{0}}{\left(D-p_{0}\right)}\frac{1}{\sqrt{n}},$$ (7)

Where D is the number of predictors, n is the number of samples, and *p*_0_ represents the least integer value of 10% of the number of predictors. In summary, the horseshoe prior allows some coefficients to be completely unregularized thereby allowing some traits to be strongly associated with a variant of interest while the coefficients of weakly or unassociated traits are shrunk toward zero (Piironen and Vehtari, 2017c).

In addition to the horseshoe prior, we provide code for execution of Bayesian ridge and lasso models to compare results and provide additional options for analysis. Ridge priors follow a traditional normal distribution with zero mean and variance of five. Similarly, the lasso model may be described as identifying the posterior mode for a Gaussian likelihood when the coefficient priors have independent Laplace distributions. This model uses a tuning parameter with a chi-squared prior with an expected value of one to determine the value of lambda for coefficient shrinkage. The Laplace distribution is then characterized by a zero mean and model-tuned variance. Significant features were determined based upon parameter estimates where the 95% central (quantile-based) posterior interval estimates from MCMC draws did not overlap zero. Posterior intervals were plotted for each variant using bayesplot (Gabry et al., 2019; Gabry and Mahr, 2022).

### 2.4 Processing and modelling feature data

Variant data used in the BGPWAS model were selected from the full set of InDels using ranges for known loci and novel associations in concert with BEDTools (Quinlan and Hall, 2010) or BCFtools (Danecek et al., 2021). Any features overlapping those ranges were written to gene-specific VCF files before converting variant data to numeric format using VCFtools (Danecek et al., 2011). Gene-specific variants were iterated over using BGPWAS to identify significant features, where iteration, modelling, and plotting were all done in R version 4.1.0 (R Core Team, 2021). Annotation information was obtained from the sorghum BTx623 version three annotation (McCormick et al., 2018), UniProt (UniProt Consortium, 2021), and/or the String protein-protein interaction database (Szklarczyk et al., 2019). Individual String networks were generated using either sorghum or rice genes to identify interacting proteins and functional pathways. These networks may further be used to identify putatively interacting proteins subject to AlphaFold modeling. Scripts used for analyses are available on GitHub (https://github.com/jlboat/BGPWAS).

### 2.5 Protein alignment and folding

InDels with significant associations for Ma1, Tan1, and Dw2 were then used in conjunction with the corresponding transcript sequences to manually generate the mutant alleles. Alternative transcripts were then translated using ExPASy (Gasteiger et al., 2003) to determine the effects of each variant on protein sequence. The resulting proteins were aligned with the annotated transcripts using the Clustal Omega multiple sequence aligner through EMBL-EBI with default parameters (Madeira et al., 2022) to visually compare the original and truncated sequences. Protein folding was performed for alternative transcripts using AlphaFold 2 (Jumper et al., 2021) through ColabFold (https://github.com/sokrypton/ColabFold) (Mirdita et al., 2022) available through Google Colab (https://colab.research.google.com/). AlphaFold two was executed using the global superposition metric template model (TM) for protein structure prediction. Output from AlphaFold two includes PDB formatted structures sorted by average predictions of side-chain *χ* angles and per-residue accuracy of the structure estimated using the predicted local distance difference test (pLDDT) where complexes are sorted by the predicted TM score, plots of the model quality (i.e., predicted aligned error (PAE), pLDDT, and sequence identity to query coverage), and multiple sequence alignment files (Mirdita et al., 2017; 2019; Mitchell et al., 2019; Jumper et al., 2021; Mirdita et al., 2022).

## 3 Results

### 3.1 Bayesian model comparison

**FIGURE 1:**
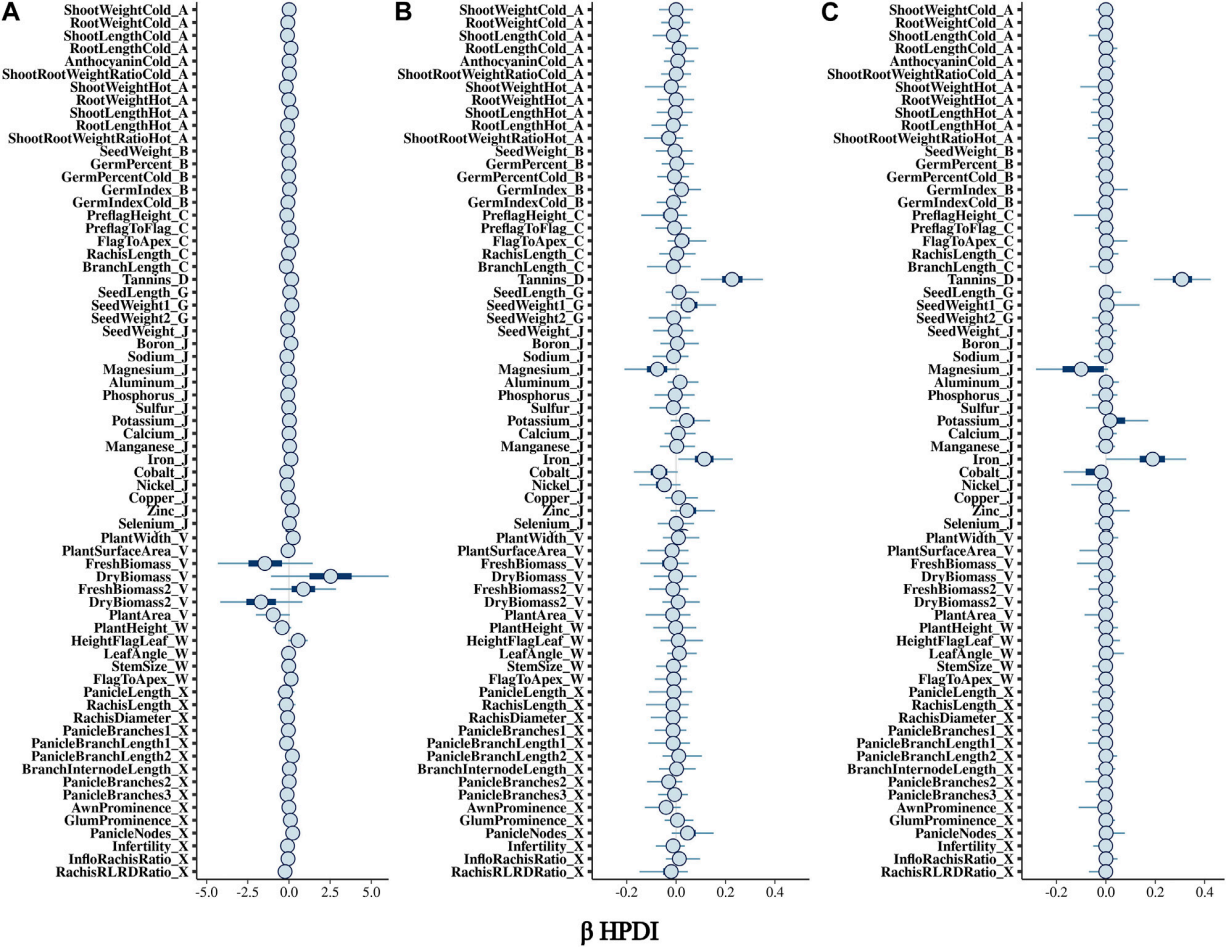
Posterior estimates of *β* for traits associated with Tan2 (Chr2:7,976,118) using **(A)** Bayesian Ridge, **(B)** Lasso, and **(C)** Horseshoe Regression. Boxplots represent the 95% highest posterior density interval (HPDI) based on MCMC samples.

**FIGURE 2:**
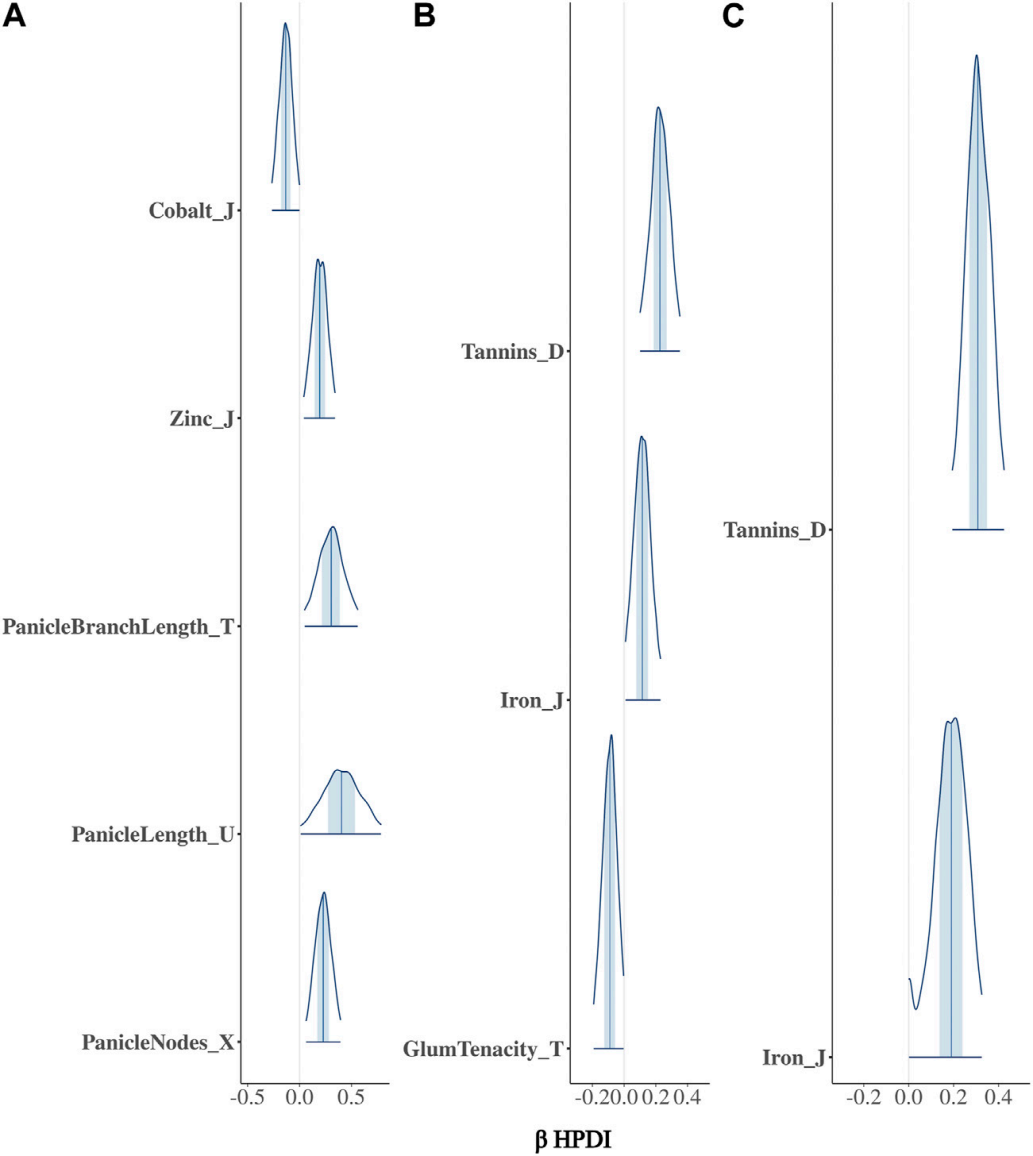
Posterior estimates of *β* for traits significantly associated with Tan2 (Chr2:7,976,118) using **(A)** Bayesian Ridge, **(B)** Lasso, and **(C)** Horseshoe Regression. Histograms represent the 95% highest posterior density interval (HPDI) based on MCMC samples.

The BGPWAS was designed using a horseshoe prior to effectively shrink non-relevant coefficients (Piironen and Vehtari, 2017c). This allows for feature selection across the phenomic data while permitting estimation of the remaining parameters without the strong shrinkage. By comparing Bayesian Ridge regression results (Figures 1A, 2A) with those from the Lasso (Figures 1B, 2B) and horseshoe model (Figure 1C and 2C), it is clear that the horseshoe and Lasso priors effectively shrink priors of non-relevant traits as compared to the Ridge model (Figure 2C). The Ridge regression incorrectly associated several panicle-related traits with the tannin-related locus, which was a consistent pattern across Ridge regression results for different loci. Unlike Ridge regression, Lasso results were largely consistent with the traits known to be associated with characterized loci. However, the Horseshoe regression provided unshrunken estimates of beta, thereby leading to stronger associations than those of the Lasso model. As the Horseshoe regression results were largely similar with the exception of the shrinkage differences, only the variants that showed strong associations from Horseshoe regression were considered for downstream loss-of-function analyses.

### 3.2 Confirmation of characterized loci

In sorghum, there are several well-characterized loci for maturity (*Ma1* and *Ma3*), tannin content (Tan*1*, Tan*2*, and *Y1*), and plant height (*Dw1*, *Dw2*, and *Dw3*). We performed BGPWAS analysis of InDels falling within these genes as a means of assessing model accuracy. For maturity genes, many maturity-dependent traits were associated - particularly traits mediating both biomass and grain yield (Table 1). Similarly, the dwarfing genes were strongly associated with plant height traits such as the stem size, length from flag leaf to apex, and plant surface area (Table 1). Genes *Ma1* and *Dw2*, which are known to have large, pleiotropic effects, had a higher number of associated traits than those genes that only affected a few traits. The pleiotropic nature of several tannin genes was also evident as three of the primary tannin loci were also associated with metal binding (aluminum, iron, and zinc) (Figures 1, 2; Table 1). The affinity of tannins for metal binding is well documented across a variety of metals and plant species including close relatives of sorghum, maize, and pearl millet (Kidd et al., 2001; Barcelo and Poschenrieder, 2002; Kochian et al., 2004; Lestienne et al., 2005; Su et al., 2022).

**TABLE 1:** Genotype-Phenotype associations by gene.

Gene name	Gene ID	Phenotypes
*Characterized*		
*Tan2*	Sobic.002G076600	Tannins, Iron
*Tan1*	Sobic.004G280800	Tannins, Zinc
*Y1*	Sobic.001G397900	Aluminum
*Dw1*	Sobic.009G229800	PlantSurfaceArea
*Dw2*	Sobic.006G067700	FlagToApex, PanicleBranches,PanicleBranchLength
*Dw3*	Sobic.007G163800	StemSize
*Ma1*	Sobic.006G057866	HeightFlagLeaf, HydrolysisRate,LeafAngle, ShootLength,ShootWeight
*Ma3*	Sobic.001G394400	PanicleBranches, Tannins
*Uncharacterized*		
*Dw4*	Sobic.006G028000	BranchInternodeLength
*HEAT*	Sobic.003G266100	Tannin, Aluminum,SeedWeight, SeedDeterioration*etc.*
*CYP711A1*	Sobic.003G269600	Tannin, SeedWeight
*CMT*	Sobic.003G269700	Tannin, Copper,FlagToApex
*MYB86*	Sobic.003G270300	Tannin, SeedAcidFiber,SeedWeight, Sodium*etc.*
R locus	Sobic.003G233200	Tannin, Zinc,BranchInternodeLength
CA	Sobic.003G234200	Tannin, Aluminum,RachisTraits*etc.*
UBC	Sobic.001G526600	Indium
PLATZ	Sobic.007G018550	BranchInternodeLength, BranchLength,RachisLength

**FIGURE 3:**
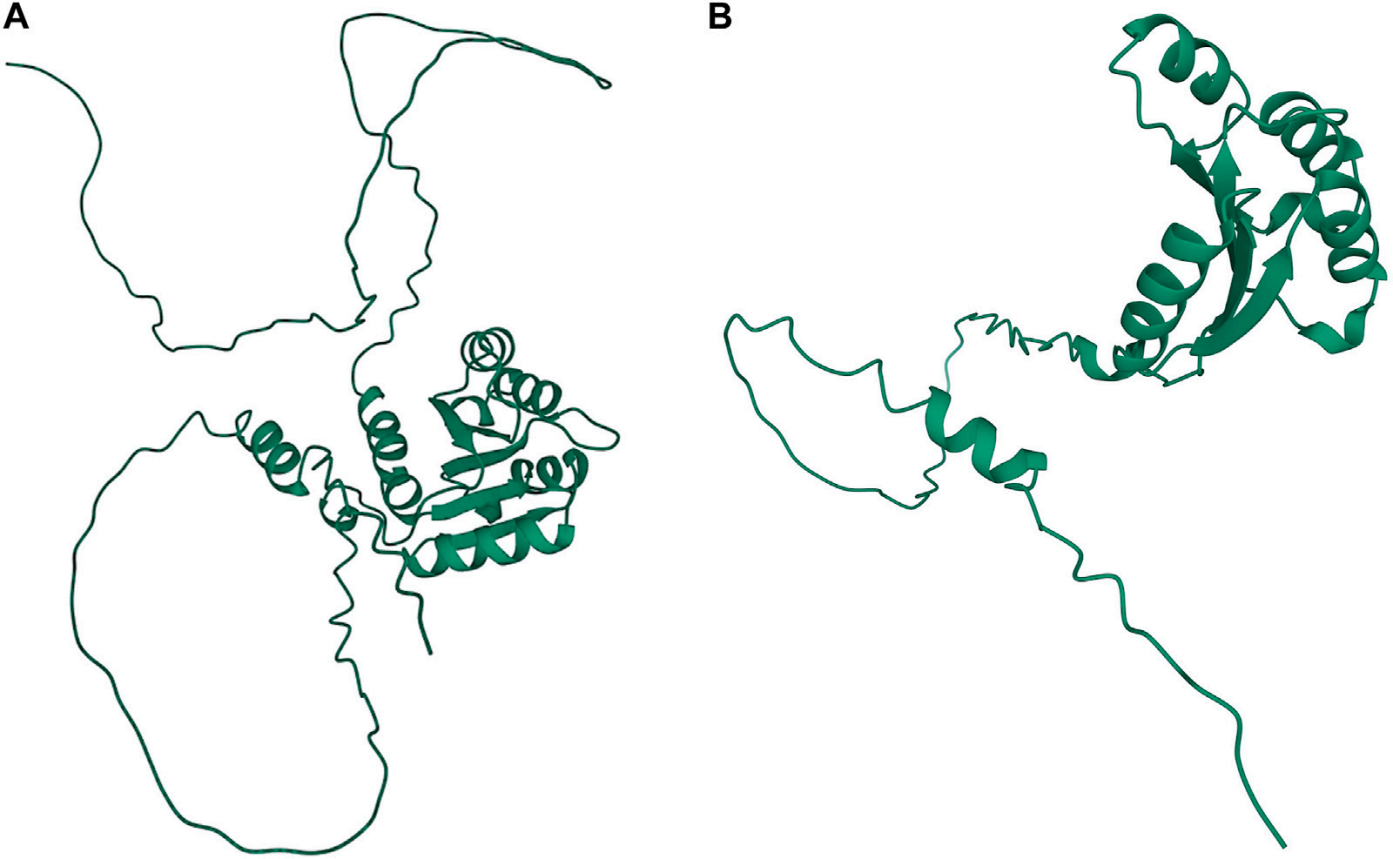
Protein structures predicted for Ma1 using AlphaFold two for both the **(A)** annotated protein and **(B)** truncated protein based on a single-base deletion at Chr06_40312436. Protein coils represent *α* helices and arrows represent *β*-pleated sheets.

**FIGURE 4:**
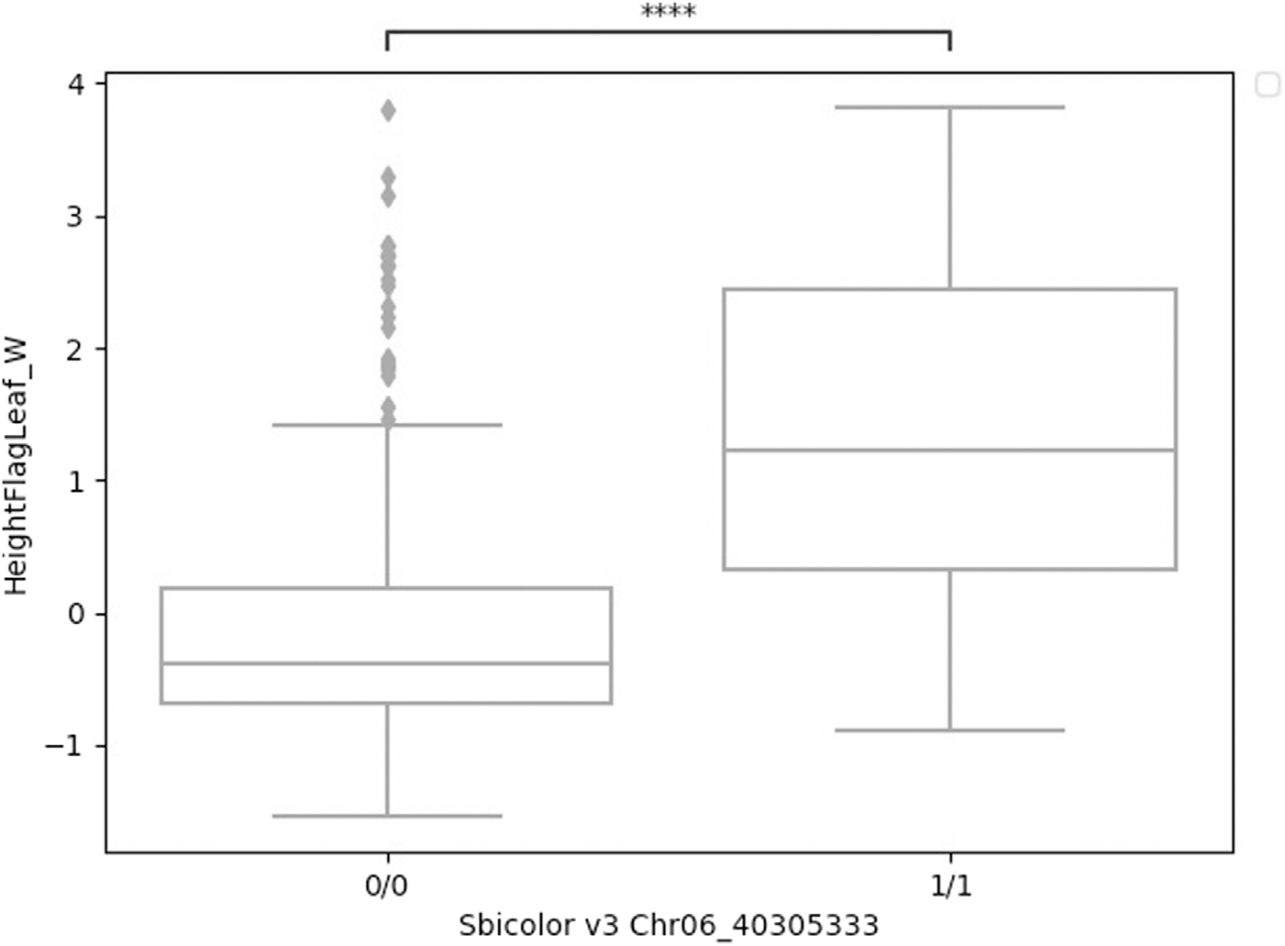
Boxplots of height to flag leaf best linear unbiased predictions by genotype (either homozygous reference 0/0 or homozygous alternative 1/1) for a four-base deletion in *Ma1* at Chr06_40305333. These two groups were significantly different (*t*-test) at p < 0.001.

**FIGURE 5:**
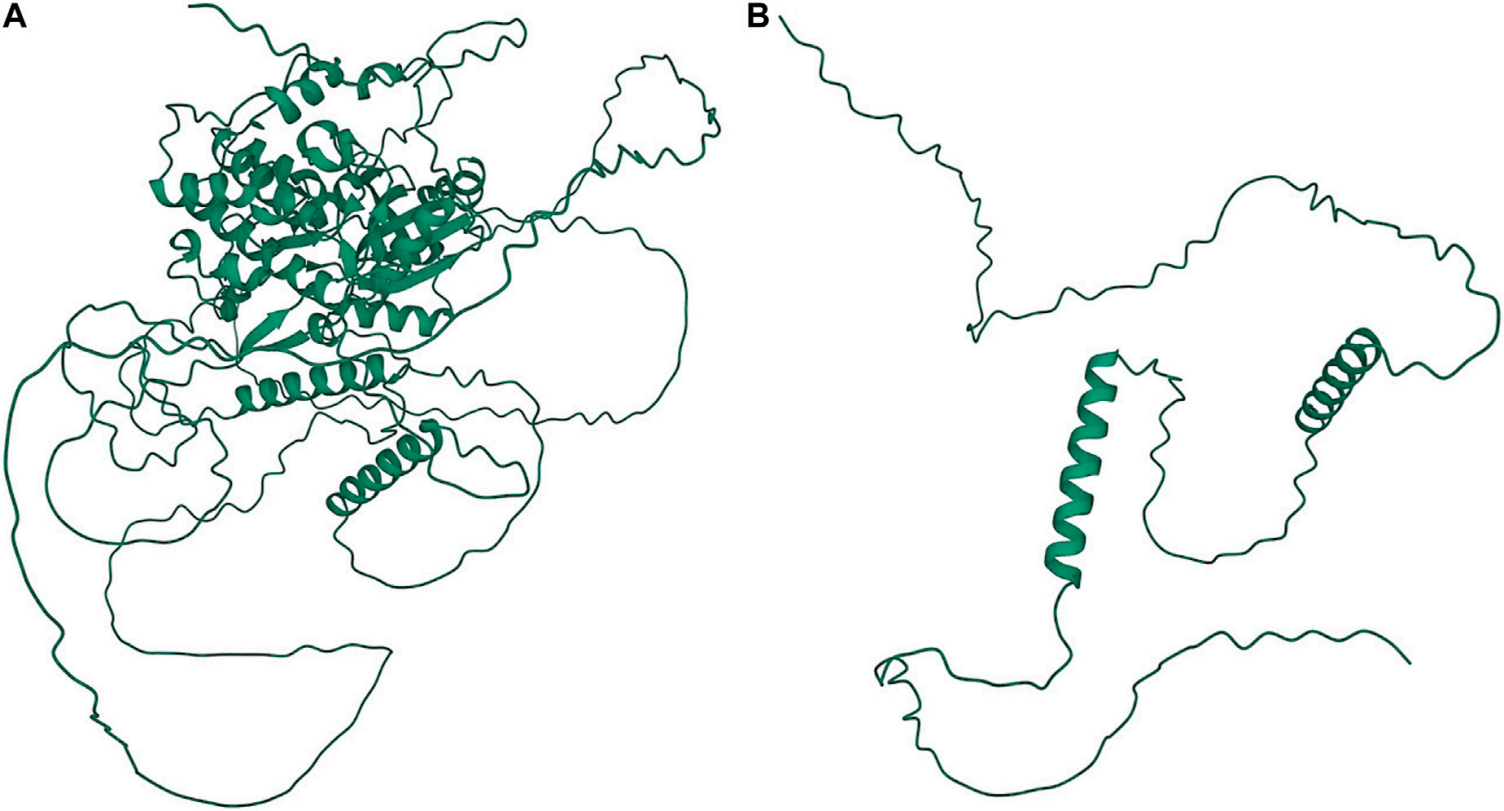
Protein structures predicted for Dw2 using AlphaFold two for both the **(A)** annotated protein and **(B)** truncated protein. Protein coils represent *α* helices and arrows represent *β*-pleated sheets.

A significant association in *Ma1* with a single-base deletion at Chr06_40312436 corresponded with a loss-of-function mutation that affects the protein product *via* a frameshift mutation (Figures 3, 4). The *ma1* mutant protein is approximately 60% the size of the native transcript. This truncation also results in the partial loss of a response regulatory domain and the total loss of an intrinsically disordered region (IDR). Similarly, for the well-documented *Dw2* gene, we identified a loss-of-function mutation that significantly affects the translated protein *via* a frameshift mutation that results in a product less than one-quarter the original size (Figure 5). This frameshift mutation also results in the loss of the sole kinase domain as well as three IDRs (UniProt Consortium, 2021).

**FIGURE 6:**
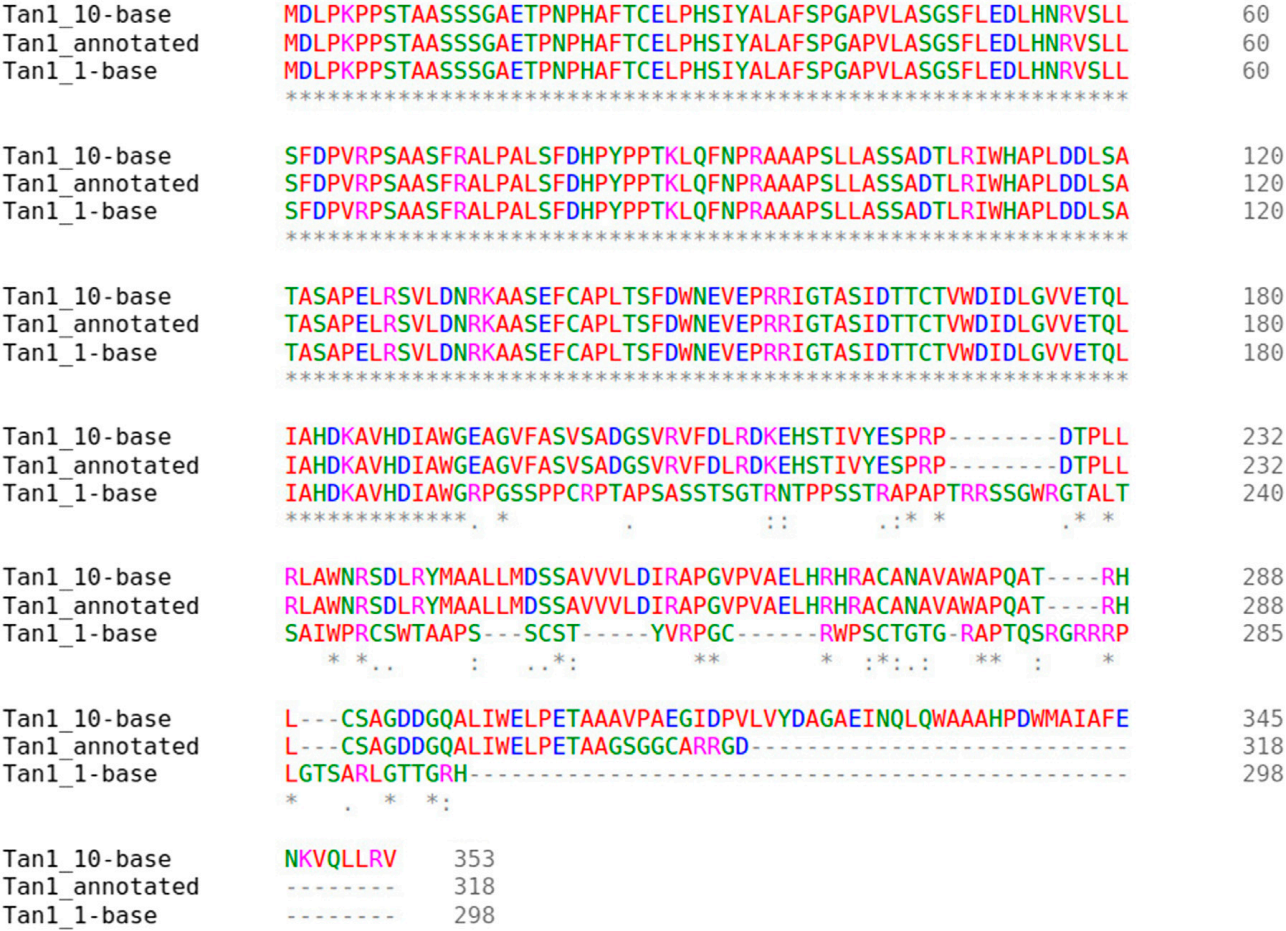
Multiple sequence alignment of the *Tan1* alleles. The allele names include the *Tan1* allele with a 10-bp deletion (Tan1_10-base), 1-bp deletion (Tan1_1-base), and the BTx623 version 3.1.1 annotated protein (Tan1_annotated). Consensus symbols include asterisks, which indicate positions with fully conserved residues, colons, which indicate conservation among amino acids with strongly similar properties, and periods, which indicate conservation between groups with weakly similar properties. Positions without consensus symbols represent non-conserved substitutions or deletions.

**FIGURE 7:**
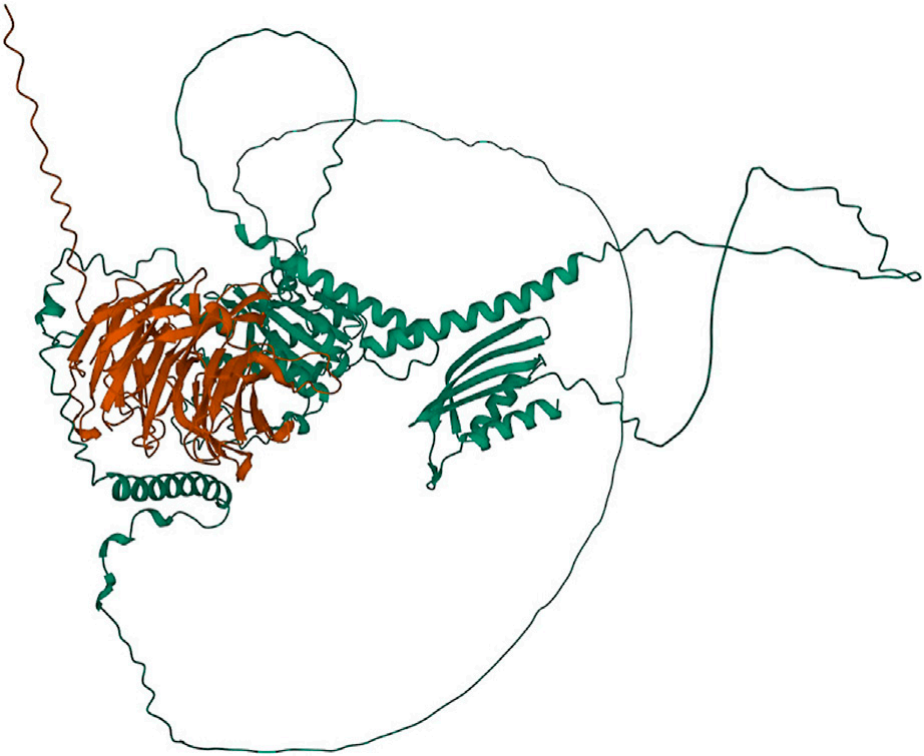
Protein structures predicted for the Tan1-Tan2 multimer using AlphaFold two for the translated *tan1b* allele and the Tan2 annotated protein. Protein coils represent *α* helices and arrows represent *β*-pleated sheets.

Two major alternative alleles were identified within the *Tan1* gene (Figure 6). The two alleles identified were consistent with the previously identified *tan1a* and *tan1b* alleles (Wu et al., 2012) with the exception that the 10-bp deletion sequence (deletion relative to the BTx623 reference genome in contrast to Wu et al. (2012) where it was considered an insertion relative to the ShanQuiRed wildtype—PI656025) was GCGGCGGGCA instead of CGGGCAGCGG. This difference may occur due to reference genome versions, the different sequencing approaches used, or a technical error. The annotated Tan1 protein exhibited predicted multimer formation scores with Tan2 at the threshold for acceptable interface pTm (ipTm) scores (ipTm > 0.7), where an ipTm of zero indicates no evidence of multimer formation, an ipTm of one represents a perfect score, and 0.75 represents a standard significance cutoff. Translation of the *tan1a* mutant allele resulted in a protein product that demonstrated exceptionally poor predicted multimer formation scores (ipTm < 0.3) with the annotated Tan2 protein (Figure 7). These results contrast with those seen for the 10-base altered *tan1b* allele, which demonstrated very high predicted multimer scores (ipTm > 0.9). This strong interaction effect is also consistent with the fact that mutations in either gene have been shown to mask effects of a dominant allele at the other locus (Yang et al., 2022).

### 3.3 Candidate prediction for uncharacterized loci

**FIGURE 8:**
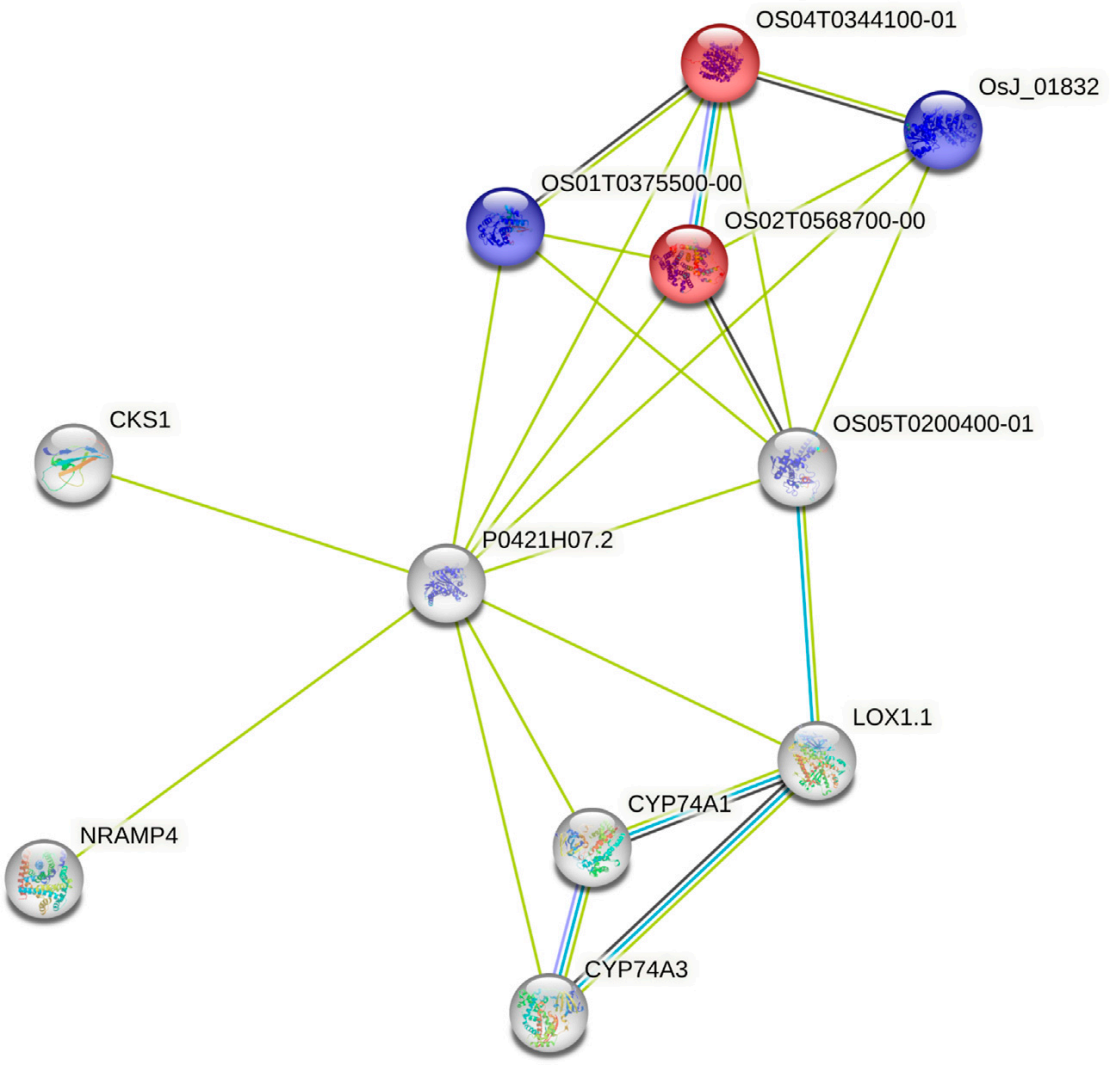
Protein-Protein Interaction Network for the rice ortholog (P0421H07.2) of Sobic.003G269700. Interaction types were represented by different colored edges between nodes that represent genes. Functional KEGG pathways involved in terpene synthase activity (red) and the Shikimate metabolic process (blue) are highlighted. Network results were generated using the String protein-protein interaction database (Szklarczyk et al., 2019).

**FIGURE 9:**
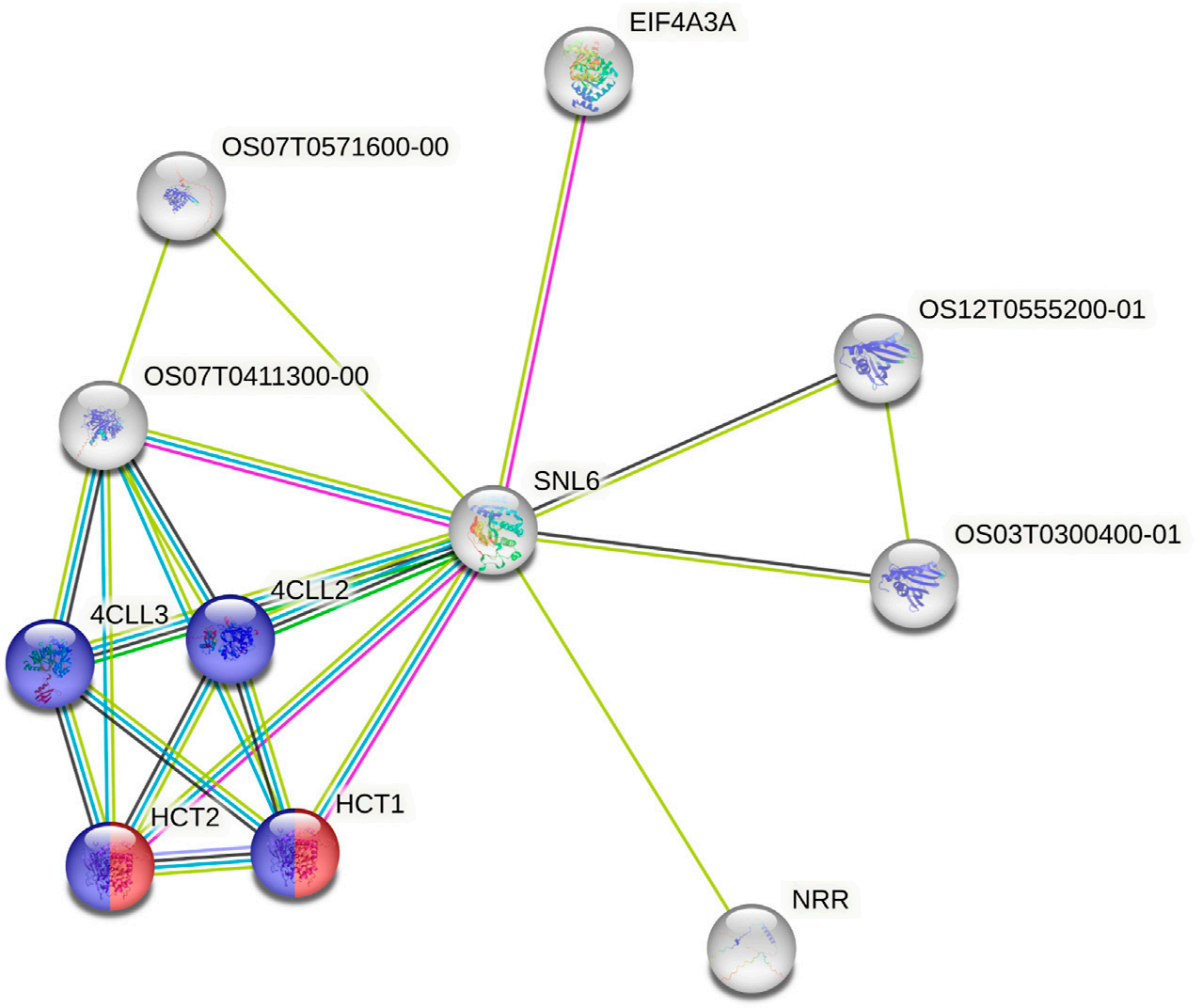
Protein-Protein Interaction Network for the rice ortholog (SNL6) of the R locus gene Sobic.003G233200. Interaction types were represented by different colored edges between nodes that represent genes. Functional KEGG pathways involved in flavonoid (red) and phenylpropanoid (blue) biosynthesis are highlighted. Network results were generated using the String protein-protein interaction database (Szklarczyk et al., 2019).

Using the data from the WGS of the SAP (Casa et al., 2008; Boatwright et al., 2022a), we selected 46 genes falling around the novel loci identified by Boatwright et al. (2022a) for tannin content 100 kb upstream and downstream of the top associations on Chr03 (centered at Chr03:60,368,179 and Chr03:60,722,769) as well as 64 genes within a larger span from 57 to 57.5 Mb on Chr03, which were selected based on previous mapping of the R locus (Rhodes et al., 2014; Kimani et al., 2020; Nida et al., 2021). This resulted in the selection of 110 genes for subsequent BGPWAS analysis. From the set of 46 genes, only four exhibited associations with tannin content including Sobic.003G266100 (a HEAT repeat-containing protein), Sobic.003G269600 (a cytochrome P450 gene, CYP711A1), Sobic.003G269700 (a SAM-dependent carboxyl methyltransferase), and Sobic.003G270300 (MYB86). CYP711A1 orthologs like that at Sobic.003G269600 are known to be involved in strigolactone/carotenoid biosynthesis (Vinde et al., 2022). Similarly, the sorghum gene Sobic.003G270300 encodes a MYB transcription factor, and orthologs of this gene are known to regulate flavonoid biosynthesis (Cheng et al., 2021; Song et al., 2022). In rice, the Sobic.003G269700 ortholog (Os01g0701700) is closely associated with genes involved in terpene synthase activity (red) and the Shikimate metabolic process (blue) (Figure 8), which serves as the gateway to biosynthesis of phenylpropanoids (Tian et al., 2019). Similarly, from the set of 64 genes representing putative R locus genes, only two genes were associated with tannins, Sobic.003G233200 and Sobic.003G234200. Another gene, Sobic.003G270500, was previously identified as a potential candidate gene for the R locus (Boatwright et al., 2022a). While this locus encodes a farnesyl diphosphate transferase known to regulate terpene and terpenoid biosynthesis (Figure 9) (Davis and Croteau, 2000; Boatwright et al., 2022a), we did not detect any significant associations for tannin content. Instead, based on BGPWAS results, the two candidate genes were Sobic.003G233200 and Sobic.003G234200. Sobic.003G233200 encodes a cinnamoyl-CoA reductase-related (CCR-related) gene, and Sobic.003G234200 encodes a carbonic anhydrase.

In addition to tannin related loci, we also explored the QTL known as *Dw4* for the best candidate gene. The *Dw4* locus has been previously identified by multiple publications (Morris et al., 2013; Miao et al., 2020; Boatwright et al., 2022a). Using variants 100 kb upstream and downstream of the QTL on Chr6, we performed BGPWAS to identify potential candidate genes. BGPWAS results for the *Dw4* locus (Li et al., 2015; Boatwright et al., 2022a) indicate that Sobic.006G028000 is the primary candidate regulating plant height at this site. This gene encodes a fatty acid amide hydrolase.

## 4 Discussion

The primary methods utilized to perform phenomic analysis for genotype-phenotype associations involve individually identifying associations for every variant-trait pair one-at-a-time. Alternative methods for multiple traits include multiple response GWAS (Zhou and Stephens, 2014), meta-analyses Urbut et al. (2019), and the recently developed GPWAS (Liang et al., 2020). Among these, both meta-analyses and GPWAS provide the most potential for identifying pleiotropic effects of variants across a range of traits. Uniquely, GPWAS does not rely on traditional GWAS methodology as a precursor for the identification of pleiotropy. Similarly, GPWAS scales much more efficiently than meta-analyses, which incorporate estimates for every variant with every additional trait included. As the scale of sequencing data increases, meta-analyses will grow increasingly computationally prohibitive. As such, we assess the application of a BGPWAS for the identification of pleiotropy and to serve as a means of high-throughput characterization for previously uncharacterized loci.

### 4.1 Benefits and limitations of Bayesian modelling

Liang et al. (2020) performed GPWAS using a forward stepwise regression. In that study, every model converged within 35 iterations. However, the authors also noted that further assessment was necessary to determine how well the GPWAS model would converge with varying numbers of individuals or traits, especially when highly correlated. Here, we remove highly correlated phenotypes based on a Pearson’s correlation coefficient > 0.95, and the implementation of the NUTS allows for reduced sensitivity to correlated parameters (Hoffman and Gelman, 2014). Further, our model permits the designation of a user-defined *τ* to control the effective number of expected non-zero coefficients. Stepwise regression, while an efficient approach for identifying potential features in a regression model, exhibits several shortcomings that are exacerbated with big data (Smith, 2018). Stepwise regression performs what is essentially local optimization of features by including explanatory variables incrementally. As such, some real explanatory variables with causal effects may not be statistically significant, and conversely, some nuisance variables may be significant. Similarly, the inclusion of nuisance variables risks collider bias among the covariates in the reverse regression (Day et al., 2016). Instead, the shortcomings of stepwise regression are better addressed using Bayesian approaches where feature effects have explicit prior uncertainty that mediate the relevance of included features (Smith, 2018) and where parameters may be estimated in an unbiased manner when the response variable is not conditionally independent of one explanatory variable given another explanatory variable (Kelter, 2022). Since Bayesian models provide several benefits over frequentist stepwise regression, we have opted to compare the effectiveness of Bayesian models for variable selection including ridge, lasso, and horseshoe priors (Piironen and Vehtari, 2017a; b,c). Both the lasso and horseshoe priors are examples of sparse Bayesian models, which induce variable selection by shrinking coefficients to zero. These sparse Bayesian models are able to estimate parameters in which *p* ≫ *n* (Johnson, 2013).

Limitations for the BGPWAS include longer runtime requirements than frequentist approaches and, in its current form, variants must be run individually - rather than running a multiresponse model. The NUTS generates estimates for a single variant in about 3 minutes (including data import and cleaning), which can be a limiting factor at larger scales. We minimized computational requirements both by focusing analyses on known QTL and by limiting associations to InDels that resulted in frameshift mutations. This not only reduces the total number of tests necessary but also shifts focus to those InDels most likely to result in loss-of-function mutations. Similarly, the BGPWAS was not written in multi-response form both to reduce computational requirements and avoid complications in sampling. Sampling of multi-response models resulted in a significant number divergent transitions and significantly longer sampling times to overcome low effective sample sizes. Instead, running variants individually allowed for parallelization of runs and circumvented issues with highly correlated variants, which are exceptionally common when examining variants falling within a single locus. Importantly, issues with generating estimates from complex, multi-response models are not unique to Bayesian models, but rather the Bayesian model makes explicit the issues inherent in generating estimates from complex models that derivative-based maximum likelihood approaches do not.

### 4.2 In-silico characterization of maturity and dwarfing loci

For validation of our model, we primarily focused on two well-documented traits, plant height and tannin content. Not only are these traits thoroughly studied in sorghum (Rhodes et al., 2014; Li et al., 2015; Boatwright et al., 2022a), but these traits are also the focus of studies across plant models (Yin et al., 2022). Genes regulating both maturity and plant height were introgressed into elite germplasm during the sorghum conversion program (SCP) for photoperiod conversion and short stature (Stephens et al., 1967; Duodu et al., 2003; Klein et al., 2008). These traits represent vital characteristics for sorghum grain production in temperate environments and combine harvesting. Similarly, tannins provide value in reducing bird pressure and human gut health but confer a bitter flavor to the grain and impede protein digestibility *via* indigestible protein–tannin complexes (Tipton et al., 1970; Yang et al., 2022). In addition to focusing on genes important for breeding targets, we also focus discussion on InDels with significant effect on protein structure. To date, studies on InDels are limited in plants, particularly in regard to their application in a breeding program. However, InDels represent an important class of variants that are more likely to affect protein structure or function when they occur within an open reading frame. Further, many programs focus on the use of SNP variants due to the ease of obtaining such data and the scale at which they may be acquired. We highlight the flexibility of our model to incorporate diverse variant types for discovery.

The mutant alleles of the *Ma1* gene were introgressed into sorghum breeding lines during the SCP due to their ability to confer early maturity. Ma1 has several known loss-of-function alleles that have been validated using positional cloning (Murphy et al., 2011). One particular InDel identified in this gene, previously identified as *Sbprr37-1* (Murphy et al., 2011), significantly affects protein length (a 40% reduction in length) *via* a frameshift mutation that results in an early stop codon. This early truncation also results in the loss of an IDR and an response regulatory (RR) domain. IDRs increase the functional versatility of proteins by facilitating interactions between the structural domains of other proteins, and IDRs are frequently targeted for post-translational modifications that affect the functional state of the protein (Van Der Lee et al., 2014). Response regulatory domains are known to interact with phosphorylated histidine kinases and catalyze the transfer of a phosphoryl group to an Asp residue in the protein containing the RR domain. RR domains also demonstrate the ability to catalyze autodephosphorylation and regulate effector domain activity in a phosphorylation-dependent manner (Gasteiger et al., 2003). As over half of the response regulatory domain is lost, this frameshift mutation results in a non-functional protein as previously determined using cloning (Murphy et al., 2011). As *Ma1* affects plant maturity, it also exhibits a pleiotropic effect across a variety of traits such as height to flag leaf as identified here (Figure 4).

Among the known dwarfing genes in sorghum (*Dw1*-*3*), we were able to identify a loss-of-function mutation in *Dw2*. Along with the other major dwarfing genes, *Dw2* was introgressed into breeding lines during the SCP due to its ability to confer short stature which facilitates combine harvesting. This particular gene exhibits a large effect on plant height and was the second most significant QTL identified for plant height based on whole-genome sequencing of the SAP (Boatwright et al., 2022a). As the InDel located within *Dw2* results in significant loss of several IDRs and a kinase domain, this frameshift mutation likely results in complete loss of function. In addition to the known Dw loci, we also explored the genes falling within a known QTL (on Chr6) designated as *Dw4* (Morris et al., 2013; Miao et al., 2020; Boatwright et al., 2022a). Importantly, this locus is not to be confused with another QTL for plant height and biomass, which has also been categorized as a potential *Dw4* locus (on Chr4) (Li et al., 2015; Brenton et al., 2020; Boatwright et al., 2022b). The only significant association for plant height within the documented QTL was Sobic.006G028000, which encodes a fatty acid amide hydrolase. Interestingly, plant amidases have been shown to serve important physiological roles in plant growth and stress responses (Moya-Cuevas et al., 2021). The Arabidopsis ortholog for this amidase gene exhibits some role in sink-to-source transition within the vascular tissues (Wu et al., 2013). Sink-to-source transition represents a vital breakpoint in development at which carbon and nitrogen pools are remobilized, and there is a transition to accumulating carbohydrates and depleting both inorganic and organic nitrogen (Masclaux et al., 2000). As such, this gene may regulate plant height by prolonging the growth phase of sorghum plants and delaying grain filling and senescence (Masclaux et al., 2000).

### 4.3 In-silico characterization of tannin loci

In Arabidopsis, there are three transparent testa genes known to regulate tannin content, including *TTG1* (a WD-repeat protein), *TT2* (an R2R3-MYB transcription factor (TF)), and *TT8* (a basic helix-loop-helix (bHLH) TF). These three genes work in a complex and directly activate *BANYULS* expression (Baudry et al., 2004). However, this complex is not conserved in maize, a closer relative of sorghum. Instead, PL/C1 (MYB TFs) and B/R (bHLH TFs) proteins mediate developmental-stage- and tissue-specific patterns of anthocyanin production, while PAC1 (a WD40 protein) is required by both B1 or R1 proteins for maximum production of anthocyanin in root tissue and seeds (Carey et al., 2004). While TT2 and TT8 are believed to form a heterodimer in sorghum, the full set of interactors during potential multimer formation is unclear. We also assessed whether the sorghum TT2 (*Y1*) and TT8 (*Tan2*) exhibited significant binding affinity as that observed for the orthologs in Arabidopsis. Iteration of the various protein isoforms all resulted in ipTm values less than 0.25, well below our significance threshold of 0.75. Similarly, the multimer of *Tan1*, *Tan2*, and *Y1* exhibited ipTm values < 0.6. Given the sensitivity of Alpha Fold to the isoform used, it may be that the *Y1* allele used was incorrect for multimer formation. We additionally examined the potential for Tan1 (a WD-repeat protein like TTG1) and Tan2 proteins to generate a heterodimer. Interestingly, the *tan1b* allele exhibiting a high predicted multimer score with the annotated *Tan2* allele at an ipTm > 0.9 (Figure 7). This is potentially consistent with *Tan1* serving the homologous role of Arabidopsis *TTG1* (or maize *PAC1*) and *Tan2* as the TT8 ortholog. This interaction is consistent with observations that mutations in either gene mask effects of a dominant allele at the other locus (Yang et al., 2022). By jointly using the BGPWAS model with Alpha Fold, the process of screening functionally relevant mutations may be reduced to just a few hours of compute time. It is worth noting that there are several limitations for multimer modeling that are worth exploring. First, there are limitations to the current AlphaFold model that may make particular protein conformations difficult to model. Thus, the absence of a strong multimer model may not be indicative of the true biological state. Second, multimer formation may be rescued in spite of the presence of indels in a given gene by stoichiometry of functional monomers, isoforms, other homologues present in the sorghum genome. Running additional models with potential functional homologs could support a redundant action. Though, prediction of protein conformation is currently the most resource intensive step of this current approach and likely will remain so in the near future. Non-etheless, where multimer formation (or lack of formation) is supported by significant phenotypic differences and BGPWAS associations between wild type and mutation individuals, these *in silico* methods may provide significant support prior to exploring tissue culture or transformation.

While the R locus represents a regularly identified locus for tannin content, to date, the gene responsible for these associations has remained elusive. In our analysis of the variants falling within this QTL, we identified two primary candidates, Sobic.003G233200 and Sobic.003G234200. Sobic.003G233200 encodes a cinnamoyl-CoA reductase-related (CCR-related) gene. Significantly, CCR genes have been shown to profoundly affect soluble phenolic pools in tomato (Van der Rest et al., 2006), and both the Arabidopsis and rice orthologs of this gene are associated with flavonoid (red) and phenylpropanoid (blue) biosynthesis (Figure 9) (Szklarczyk et al., 2019). Similarly, CCR genes affect traits similar to those known to be regulated by the R locus in sorghum, particularly where knockouts exhibit reduced tannins and a yellow seed color in *Brassica napus* (Yin et al., 2022). Conversely, the only other gene associated with tannin content within the R locus span was Sobic.003G234200, which encodes a carbonic anhydrase. As tannins are known to act as carbonic anhydrase inhibitors through inhibitive binding by two tannin molecules (Karioti et al., 2016), this action does justify the association. However, this action does not support the functional role associated with known phenotypes for this locus and as such, this gene is not likely to significantly affect seed color nor to be the primary driver for associations within the R locus. The ability to further dissect the role of individual genes within a QTL has the potential to improve breeding pipelines whether through targeted breeding (Zhang et al., 2014), marker-assisted selection (Dudley, 1993), or another technology.

### 4.4 Additional examples of model accuracy

As an additional example of model accuracy, we assessed a random selection of 30 genes - three per chromosome - representing 322 variants - both SNPs and InDels. In total, 10 of the 30 genes had no associations, but the remaining 20 genes were associated with 65 traits. Here, we highlight some interesting associations. One of the gene examined was the sorghum gene Sobic.007G018550 (Table 1). While this locus was not annotated in the original BTx623 v3.1.1 annotation (McCormick et al., 2018), later annotation of this locus (UniProt Consortium, 2021) identified it as a putative PLATZ transcription factor (TF) (Fu et al., 2020). Interestingly, PLATZ TFs are known to regulate seed endosperm development and increase the rate and duration of cell proliferation especially in the leaf tissue during earlier stages of development (Fu et al., 2020). Consistent with this, our BGPWAS identified branch length, branch internode length, and rachis lengths as the primary traits associated with this locus. We also identified a potentially novel association for a ubiquitin-conjugating enzyme (Sobic.001G526600) with indium stress. This is interesting as an Arabidopsis ubiquitin-conjugating enzyme (PHOSPHATE2: PHO2) has been shown to be required for the degradation of PHO1 protein and subsequent mediation of indium toxicity (Chang et al., 2020). By performing BGPWAS across known QTL and arbitrary genes, we have demonstrated the ability of this model to detect genotype-phenotype associations that support previously observed biological roles, identify pleiotropic effects of loci, provide functional annotation of InDels and other variants, and, in conjunction with Alpha Fold, to provide an *in silico* alternative to the traditional methods for phenotypic characterization.

## 5 Conclusion

Here, we demonstrated the value of our BGPWAS as a proof of concept approach to identify breeding targets in the form of genes with gain/loss of functions for given traits and to identify putative pleiotropy of associated loci/variants. We further show that even previously characterized sorghum genes possess major InDels that directly affect protein folding and interactions during multimer formation. As a high-throughput approach for *in silico* characterization of loci, this model could serve to expedite the process of moving from novel QTL to their functional characterization and the introgression of desired loci in a breeding program. Further, by serving as a quick *in silico* alternative to existing cloning and validation procedures, this model may serve as a vital tool for identifying key functional targets to act upon for improvement across all species and alleviate some of the current bottlenecks in functional genomics.

## Data Availability

The original contributions presented in the study are included in the article/supplementary material, further inquiries can be directed to the corresponding author.
